# Human cold pain: a randomized crossover trial

**DOI:** 10.1097/j.pain.0000000000003503

**Published:** 2024-12-17

**Authors:** Felix J. Resch, Stefan Heber, Farzin Shahi, Manuel Zauner, Cosmin I. Ciotu, Andreas Gleiss, Sabine Sator, Michael J. M. Fischer

**Affiliations:** a Center for Physiology and Pharmacology, Medical University of Vienna, Vienna, Austria; b Center for Medical Data Science, Medical University of Vienna, Vienna, Austria; c Division of Special Anesthesia and Pain Medicine, Department of Anesthesia, Intensive Care and Pain Medicine, Medical University of Vienna, Vienna, Austria

**Keywords:** Cold pain, TRPM8, TRPA1, Na_V_1.7, Na_V_1.8, Randomized controlled trial, Human pain model, Sensory neurons, Pain threshold

## Abstract

The mechanism causing cold pain in humans is unresolved. Animal data suggest a nonredundant contribution to cold pain for transient receptor potential channels TRPM8 and TRPA1 for detection and voltage-gated sodium channels Na_V_1.7 and Na_V_1.8 for conduction at these temperatures. We established an intradermal injection–based cold pain model, which allows pharmacologically addressing molecular targets at the site of cooling. Lidocaine, added to the injection solution as positive control, largely reduced cold-induced pain in 36 volunteers. The 4 mentioned molecular targets were blocked by antagonists in a double-blinded crossover trial. Pain induced by 3°C intradermal fluid was not reduced to a relevant extent by any of the 4 antagonists alone or by the quadruple combination. However, the temperature threshold for cold pain appeared shifted by the inhibition of TRPA1, TRPM8, and Na_V_1.7 and to a lesser extent by Na_V_1.8 inhibition, 4-fold inhibition decreased the threshold by 5.8°C. Further mechanisms contributing to human cold pain need to be considered.

## Abstract 2

Supplemental Digital Content is Available in the Text.

In a new human cold pain model, inhibition of transient receptor potential, TRPA1 TRPM8, and voltage-gated sodium channel, type Na_V_1.7 and Na_V_1.8, affects noxious cold thresholds but not pain from prolonged cold exposure.

## 1. Introduction

Cold-induced pain serves as a protective mechanism, warning of potentially harmful low temperatures. However, the molecular mechanisms behind cold pain remain unclear. It is important to distinguish between nonpainful cooling sensations and cold becoming uncomfortable or painful. This distinction complicates the molecular understanding of cold pain, as the definition of pain is already fulfilled by unpleasantness.^38^ Despite this conceptual complexity, and the difficulty of pinpointing the transition between cool and cold, there is a clear difference between a mild coolness and the stinging or burning sensation from extreme cold. Humans can detect small temperature drops as little as 1°C.^27^ In animals, transient receptor potential channel TRPM8 plays a key role in this detection,^42^ which may also apply to humans. However, the mechanisms leading to cold pain are far less clear, where TRPM8 and TRPA1 were suggested as detectors^22^ and voltage-gated sodium channels Na_V_1.7 and Na_V_1.8 as conductors of cold pain. Regardless of the mechanism of cold sensing, voltage-gated sodium channels (VGSCs) generate action potentials to transmit the signal, a process which is also temperature dependent. In this study, 4 ion channels, TRPM8, TRPA1, Na_V_1.7, and Na_V_1.8, were investigated for their roles in cold pain.

TRPM8-deficient animals and even more so animals lacking TRPM8^+^ neurons have reduced responses to temperatures around 0°C, but most responsiveness remains, suggesting additional cold sensors.^23^ In humans, TRPM8^+^ nerve density correlated with cold pain thresholds^47^ with a bimodal distribution.^25,29^ Systemic application of the TRPM8 antagonist PF-05105679 reduced pain in the cold pressor test, further supporting its role in cold pain sensation.^48^ In contrast to TRPM8, the role of TRPA1 in cold sensing is highly controversial.^8^ Some studies suggest that TRPA1 contributes to cold pain, particularly in pathological conditions, such as oxaliplatin-induced cold hyperalgesia, which is reduced with pharmacological inhibition or genetic knockout of TRPA1.^51^ Thermal sensitivity of TRPA1 in artificial membranes is controlled by redox reactions, with reducing and oxidizing agents affecting its activity.^34^ Compared with TRPM8-knockout mice, TRPM8/TRPA1 double knockouts show reduced cold responses, but avoidance of noxious cold persists.^49^ This is in contrast to another observation, where the double knockouts and TRPM8 knockouts showed comparable reduced cold avoidance, whereas TRPA1 knockouts exhibited no phenotype.^23^

Sodium channels, particularly Na_V_1.7 and Na_V_1.8, are essential for action potential conduction in cold conditions, where kinetics and excitability are decreased.^13,15,39^ Na_V_1.7-knockout mice have reduced cold responses,^32^ and humans with Na_V_1.7 loss-of-function mutations are completely insensitive to any type of pain, including cold pain.^30^ The slow inactivation of Na_V_1.8 is temperature independent, which enables nerve excitability also at low temperatures.^52^ Na_V_1.8-deficient animals show impaired responses to harmful stimulations in cold conditions.^33,52^

A new human cold pain model was developed and validated in this study, based on continuous intradermal injection of a cooled fluid. This allowed coinjections of specific receptor antagonists for the proposed targets. The study aimed to quantify the contribution of these targets to human cold pain.

## 2. Materials and methods

### 2.1. Experimental cold pain model

**Figure 1.**
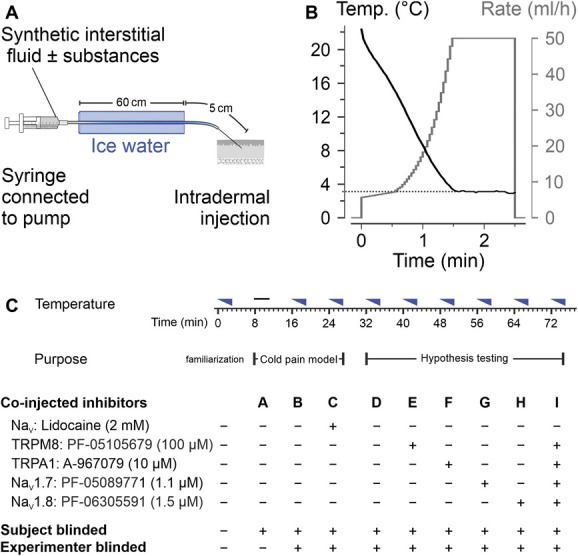
Experimental cold pain model and study design. (A) From a syringe filled with synthetic interstitial fluid ± test substances, the tubing is passed through an ice water bath. The solution equilibrates to 0°C, and the pumping rate determines the time for rewarming towards room temperature before the fluid reaches the tip of the intradermally positioned 27-gauge cannula. (B) A nonlinear increase in injection rate (grey) allows to obtain a largely linear temperature ramp at the outlet in the range 22-3°C. The volume per injection is 1242 µL. (C) Overview of the timing and type of injections. Each increasingly cold injection is indicated by a blue triangle, and the room temperature injection by a black horizontal line of equal width. The timeline below shows the approximate duration of the individual injections and the pauses in between. Below the purpose and type of injections is shown, and whether only the subject or also the experimenter was blinded. Note that each specific injection type is assigned a specific letter. A total of 36 subjects were recruited, and all completed the study. The respective sequences and the (Consolidated Standards of Reporting Trials) CONSORT flow diagram are provided in Fig. S2c, http://links.lww.com/PAIN/C192.

To expose volunteers to a defined nonhazardous intradermal cold stimulus, a method was established that reproducibly delivers a fluid with a certain temperature into the skin. The buffered interstitial fluid adapted from Bretag^6^ was used as previously described.^18^ It contained (in mmol/L) NaCl 113.8, KCl 3.5, Na_2_HPO_4_ 1.67, MgSO_2_ × 7H_2_O 0.7, sodium gluconate 9.6, glucose 5.0, sucrose 7.6, CaCl_2_ × 2H_2_O 1.5, and histidine chloride 22.0, diluted in ultrapure water obtained from a Milli-Q no plus system (MQ, Millipore, Burlington, MA). The solution was adjusted to a pH of 7.4 and filtered using a 0.2-µm filter (Sarstedt Filtropur S, 0.2 µm). This fluid, alone or supplemented with the substances of interest, was filled in a 5-mL syringe (B.Braun Injekt Luer Lock Solo, 5 mL), connected to an extension line (B.Braun Original Perfusor Line, 50 cm made from polyethylene) and that to a winged injection set with a 27-G cannula (B.Braun Venofix A, 27 G, 0.4 × 10 mm, 30 cm made from polyvinyl chloride). The syringe was inserted into a programmable pump (World precision instruments, Sarasota, FL). The tubing was immersed in an ice water bath to allow the solution to equilibrate to 0°C. The minimal immersion distance necessary to reach the lowest temperature possible (3.1°C) at the cannula tip turned out to be 40 cm for a fixed injection rate of 50 mL/hour. The length of ice water immersion was 60 cm. Between the point where the tubing leaves the ice water bath and the cannula tip, there was a fixed distance of 5 cm at which the fluid rewarms (Fig. 1A). This results in higher temperatures measured at the cannula tip compared with the temperatures measured at the end of the ice water bath (Fig. S1a, http://links.lww.com/PAIN/C192). Temperatures were measured using a thermocouple connected to a data logger (UBS-603, Measurement Computing Corporation Norton, MA). The programmable pump with preset time course delivered a total injection volume of 1242 µL. Injection rate was nonlinearly rising from 5.7 to 50 mL/hour over a period of 90 seconds and maintained at the final rate for another 60 seconds. The pumping rates after the initial phase followed a third-order polynomial. The resulting time course was measured 5-fold at the cannula tip for sequential optimization of the polynomial. The final ramp leads to a largely linear temperature decrease from initially 22.4 ± 0.7°C to a final temperature of 3.1 ± 0.1°C at the end of the ramp (Fig. 1B). The final plateau of the injection rate was added, as the pain ratings further increased in pilot experiments despite a constant 3.1°C temperature of the injection. This is likely to be explained by conduction and therefore an increasing tissue volume being cooled. With the chosen infusion protocol, it takes about 11 seconds to inject the solution located between the ice bath and the subject. The spatial temperature distribution was measured with a thermal camera (CompactPRO, Seek Thermal, Santa Barbara, CA) and shows the extent and time course of cooling over time at the skin (Fig. S1b,c, http://links.lww.com/PAIN/C192).

### 2.2. Human psychophysical experiments

The single study visit comprised 10 injections and lasted about 90 minutes (Fig. 1C). Distinct skin sites were used for testing, alternating between both volar forearms. Each injection started with insertion of the cannula superficially into the dermis of the forearms; the pain induced by this insertion was noted and used to statistically adjust for differences in pain sensitivity between skin spots. The minimum spatial distance between insertion spots was 3 cm. The pump injection protocol started when the insertion-induced pain had fully subsided. Pain was rated every 5 seconds using a numerical rating scale from 0 to 100, from the start of the injection until no pain was reported for 30 seconds. Thereafter, the cannula was removed. All subjects received a consistent and clear explanation of how to rate pain. The only verbal descriptors were “not painful,” which had to be reported as a pain rating of 0, and “painful,” for which the rest of the rating scale was reserved. A pain rating of 100 was defined as “worst pain imaginable.” Verbal descriptors of the quality of the pain (eg, burning, stinging, or aching) were not provided to avoid influencing the pain ratings.

### 2.3. Study design

A study design with a prespecified decision tree enrolled a total of 36 subjects after an internal pilot. All of these completed the protocol and were included in the analysis. The study was a single-group, randomized, placebo-controlled, crossover trial (Fig. S2, http://links.lww.com/PAIN/C192). The rationale for the crossover design was based on the following assumptions: (1) Because of generally large interindividual differences in pain perception and pain ratings in the population, it was considered advantageous to have cold-induced pain rated in each subject. A full factorial design, which would allow testing the antagonists in all possible combinations, thus consisting of 15 (excluding the control) instead of 5 antagonist combinations was not considered feasible for 1 subject. (2) Subjects were exposed to such low doses of inhibitors that only local effects at the site of cold exposure, but no systemic effects were considered plausible. This allowed several short-lasting cold stimuli ± inhibitors to be tested at different sites a few minutes apart. A potential psychological carryover effect was accounted for by a Williams design, as the pain intensity of 1 injection might influence the subsequent one.

At the beginning of the visit, an unblinded control injection of interstitial fluid with the cooling ramp (#1) was performed for familiarization with the experimental protocol and the rating scale. This was followed by 2 parts in which independent hypotheses were addressed. The first part (injections #2-4) aimed to validate the cold pain model, and the second part (injections #5-10) investigated the receptor dependence of cold pain (Fig. 1C).

#### 2.3.1. Part 1—cold pain model

This contained 1 injection without cooling of the fluid, 1 injection with cooling of the fluid, and 1 with cooling of the fluid and addition of lidocaine. The concentration of lidocaine was 2 mM, which is low compared with the 85 mM in the clinically used 1% solution, to avoid activation of TRPV1. The lidocaine injection was used as a positive control for a pain-relieving substance. The experimenter was blinded concerning which of the 2 cold injections contained lidocaine. Because the tubing needed to be immersed in ice water for cold injections, but not for room temperature injections, the experimenter was not blinded as to which of the 3 injections was the room temperature injection. The volunteer was blinded to the experimental protocol. Blinding of the volunteer for the temperature of the injected fluid was achieved by leading the injection set through the ice water bath (in a closed polystyrene box with a small outlet for the cannula) or through an identical but ice water free polystyrene box. Before each injection the volunteer had to turn around so that they could not see whether the tube with the cannula was placed by the experimenter through the polystyrene box with ice or through the one without. Thus, injections #2 to #4 were single blinded regarding the temperature and double blinded regarding lidocaine (Fig. 1C). The primary hypothesis of part 1 was that the cooled injections induce more pain than room temperature injections. The secondary hypothesis was that cooled injections are less painful in the presence than in the absence of lidocaine. Using a Williams design, the 3 treatments resulted in 6 possible sequences. Each subject was randomly assigned to one of these sequences without replacement. After a block of 6 subjects was completed, a new batch of 6 sequences was used for the next group.

### 2.4. Part 2—receptor dependence of cold pain

Every subject received 1 injection without an antagonist, 1 each with 1 of the 4 antagonists alone, and 1 with all 4 antagonists combined (Fig. 1C). Injections #5 to #10 were performed in a double-blinded manner. The 6 treatments led to another Williams design of 6 sequences. Again, each subject was randomly assigned one of these sequences without replacing it, whereby the sequence of part 1 was not taken into account, avoiding a systematic carryover from part 1 to part 2. The primary hypothesis was that addition of one of the respective antagonists reduces cold pain. The 4 secondary hypotheses were that the combined application of all 4 antagonists alone reduces cold pain.

In case a volunteer would have dropped out, the assigned sequence would have been returned into the pool of available sequences to ensure that the balanced design is achieved only with volunteers who finished the whole protocol. This procedure was repeated until the target sample size was reached. A minimum period of 2 minutes separated the last nonzero pain rating from the insertion of the cannula for the following injection. Experiments were performed by 2 male experimenters.

The study protocol prespecified all aspects provided below and was approved by the ethics committee of the Medical University of Vienna (vote 1164/2023). The study was registered at clinicaltrials.gov (NCT05935280) and was performed in accordance with the Declaration of Helsinki.

### 2.5. Outcomes

Cold pain is highly variable between the subjects, far more than, eg, heat-induced pain.^28^ As such, limited or no pain was expected during the cooling phase in a substantial fraction of subjects, leading to high intersubject variance at the beginning of the stimulation. Towards the end of the 60-second cold stimulation, a steady state of the cold distribution was assumed, and most nonzero ratings were expected, which favors detection of changes to cold pain. Therefore, as the primary outcome variable, the area under the curve of pain of the period 120 to 150 seconds (last 30 seconds of the cold stimulus, AUC Pain_3°C_) was chosen. For the room temperature injection, the same period was used. The secondary outcome variable is the area under the curve of the pain ratings over the full duration of the injection period AUC Pain_Total_. The area under the curve of the ratings was calculated using the trapezoidal rule. The outcomes were prespecified in the trial registration.

### 2.6. Interim analyses and stopping guideline

No interim analyses were planned or conducted.

### 2.7. Recruitment, participants, and setting

Participants were recruited using a notice distributed at the Medical University of Vienna. Subjects between 18 and 70 years of age and full legal capacity were eligible. To ensure that each sex was equally represented in the study population, only subjects of 1 sex were enrolled after the number of subjects of the other sex reached half of the calculated sample size. Exclusion criteria were participation in another study within the past 4 weeks, medication intake except for contraceptive drugs, drug abuse, current breastfeeding, pregnancy with test-based exclusion in all female subjects before injection #1, diagnostically verified body temperature above 38°C, known allergic diseases like asthmatic disorders or allergic skin diseases, sensory deficits, skin disease, or hematoma of unknown origin in physical examination of the test site (summarized in Table S1, http://links.lww.com/PAIN/C192). Experiments were conducted at the Medical University of Vienna in a room without physical or acoustic disturbance. Following comprehensive instruction regarding the nature, significance, impact, and risks of this study, participants provided written consent to participate in the study. Furthermore, participants were informed of the possibility to withdraw their consent for any reason at any time. In addition to the instructions given by the experimenter, participants also received an information sheet written in layman's terms, explaining the nature and purpose of the study and its procedures.

### 2.8. Applied substances

All substances were obtained from Sigma-Aldrich (St. Louis, MO), the purity of all substances was at least 98.8% based on high-performance liquid chromatography (PF-05105679: 98.8%, A-967079: 99.3%, PF-05089771: 99.5% and PF-06305591: 99.7%). Stock solutions were concentrated to 1000 times the final concentration in ethanol or DMSO. All injected solutions contained the same solvent concentration of 0.3% DMSO and 0.1% ethanol. It should be considered that there is a difference between cellular and apparent IC_50_ values in psychophysical experiments,^17^ probably because of local dilution and redistribution. Compared with cellular experiments about 6-fold higher concentration was considered for injections, because there is dilution in the extracellular space and the respective factor seemed sufficient for compensation. A-967079 was used in 3 previous trials and the respective publications.^16,17^

Antagonists might affect more than 1 target. For TRPM8 and TRPA1, we tested the effectiveness and specificity of antagonists in vitro using HEK293t cells transfected with hTRPM8 or hTRPA1, respectively. HEK293t cells grown in Dulbecco minimal essential medium (DMEM D5648, Sigma-Aldrich), supplemented with penicillin, streptomycin, and L-glutamine (1% each, all from Lonza, Basel, Switzerland) were transfected using jetPEI transfection reagent (Polyplus, Illkirch, France). The cells were then seeded on poly-D-lysine–coated black 96-well plates (∼30.000 cells/well) and incubated overnight at 37°C and 5% CO_2_. The microfluorimetry of cytosolic calcium levels was performed with calcium 6 (Calcium 6 kit, Molecular Devices, San Jose, CA) at 37°C. A pipetting fluorescence plate reader (FlexStation 3, Molecular Devices) was used, which excites every 2 seconds at 485 nm, and the area under the curve of fluorescence emission served as an index of intracellular calcium responses. TRPM8 was stimulated by WS-12, which was fully antagonized by TRPM8 antagonist PF-05105679 alone and in the presence of all 4 antagonists (Fig. S3a, http://links.lww.com/PAIN/C192). For specificity, we demonstrated that Na_V_1.7 and Na_V_1.8 antagonists did not inhibit WS-12 activation of TRPM8. TRPA1 antagonist A-967079 slightly reduced this activation at the injected concentration, but not at a 6-fold diluted concentration, reflecting the prior difference between in vitro and in vivo EC_50_ for a TRPA1 agonist in a prior study.^17^ Similarly, TRPA1 was activated by allyl isothiocyanat (AITC), which was fully antagonized by A-967079 alone and in the presence of all 4 antagonists. For specificity, we demonstrated that neither the TRPM8 antagonist nor Na_V_1.7 or Na_V_1.8 antagonists did inhibit AITC-activation of TRPA1 (Fig. S3b, http://links.lww.com/PAIN/C192). The Na_V_1.7 antagonist PF-05089771 has an IC_50_ of 11 nM; there is an at least 10-fold selectivity over all other voltage-gated sodium channels, and an at least 1000-fold selectivity over Na_V_1.8.^3^ The Na_V_1.8 antagonist PF-06305591 has an IC_50_ of 15 nM, the IC_50_ for Na_V_1.1-Na_V_1.7 and for several other targets is >30 µM.^7^

Considering potential adsorption by the plastic tubing, solutions that would have been injected from a regular cooling protocol were collected and analyzed by high-performance liquid chromatography (HPLC). Acetonitrile HPLC grade was obtained from VWR Chemicals (Fountenay-sous-Bois, France). Substances were dissolved in deionized water and quantified using RP-C18 columns (150 × 3.0 mm, 2.5 μM; Kinetex, Phenomenex, Torrance, CA), in a Dionex Ultimate 3000 UHPLC (ThermoFisher, Waltham, MA). The mobile phase was a gradient from solvent A (water with 0.1% trifluoroacetic acid) towards solvent B (10% water, 90% acetonitrile with containing 0.1% trifluoroacetic acid) with a flow rate of 0.3 mL/minute. The gradient started at 95% A/5% B and was linearly raised to 80% B over a period of 25 minutes. Absorbance was monitored at 280 nm for A-967079 and PF-06305591 or at 254 nm for PF-05105679 and PF-05089771. Results indicated a substance loss of approximately 40% for A-967079, and less loss for the other substances (Fig. S4, http://links.lww.com/PAIN/C192). To achieve the target concentrations, the loss of substance was compensated for by adjusting the concentrations of the prepared solutions. The compensation factor of 1.20 for lidocaine was determined in our prior study.^18^ The concentration of TRPA1 inhibitor A-967079 was compensated by a factor of 1.36, a factor of 1.02 for TRPM8 inhibitor PF-05105679, a factor of 1.23 for Na_V_1.7 inhibitor PF-05089771, and a factor of 1.04 for Na_v_1.8 inhibitor PF-0630559. Of note, these factors cannot be deduced from the lipophilicity given by the respective xLogP3 values of 3.2, 4.7, 4.3, and 1.5. Based on the first experiment, we adjusted the initial dose with the reciprocal of the fraction remaining in the solution after flowing through the experimental setup. The solutions with adjusted concentrations were measured again by HPLC and were close to the original planned concentration.

### 2.9. Statistical model for preplanned analyses

The distribution of AUC Pain_3°C_ values was examined for a necessary transformation. Regarding part 1 (Cold model), log-transformation of the data resulted in the best residual distribution and for the analysis of part 2, data were square-root transformed.

A linear mixed model for each study part was applied with the primary outcome variable AUC Pain_3°C_ as dependent variable. To account for the potential correlation between AUC Pain_3°C_ values from the same volunteer, a random subject factor with a AR(1) covariance pattern for part 1 and part 2 was chosen from an AR(1) (a first-order autoregressive structure with homogenous variances), a Toeplitz structure, or compound symmetry based on the lowest Akaike information criterion. To adjust for differences in cannula insertion pain between the injection spots, that is, the pain reported immediately after the cannula penetrated the skin, the respective values were included as covariate after log10-transformation in models of part 1 and part 2. To account for the crossover design, the period of the injection order was included as a categorical factor with 3 levels for part 1 and 6 levels for part 2. For the validation of the cold-induced pain model in part 1, a categorical factor with 3 levels “room temperature,” “cold,” and “cold + lidocaine” was used. To test the primary hypothesis of part 1, the AUC Pain_3°C_ of cooled and uncooled experiments was compared. To test the secondary hypothesis, the AUC Pain_3°C_ of cooled experiments without and with lidocaine was compared within the same crossover, linear, mixed model described for the primary analysis.

For the assessment of the pharmacological intervention in part 2, the predictors were the binary within-subject factors “TRPM8 antagonist,” “TRPA1 antagonist,” “Na_V_1.7 antagonist,” and “Na_V_1.8 antagonist” each with the levels “inhibitor” (substance used) and “control” (substance not used). The 4 prespecified contrasts compared each of the 4 antagonists against cold stimulation without antagonists. The secondary prespecified analysis was to compare the combination of all 4 antagonists with the expected sum of the 4 changes caused by separate antagonist application. The model parameter representing the supra- or infraadditivity was tested against 0.

Conditional residuals of each linear mixed model were inspected for gross deviations from normal distribution and leverage. All reported *P* values are the results of 2-sided tests. *P* -values of ≤0.05 were considered statistically significant. Correction for multiple testing was planned between the 4 antagonists. The primary statistical analysis was performed with SAS 9.4. Graphs were generated using R with the ggplot2 data visualization package.

### 2.10. Sample size justification and internal pilot

The effect size of pain induced by cooled vs room temperature injections was considered larger than for the pharmacological interventions. Therefore, sample size was determined for the latter. The aim was to detect a 40% reduction of AUC Pain_3°C_ in 1 of the 4 targets with a power of at least 80%. An adequate sample size was prespecified to be determined by simulations based on an internal pilot with N = 12, without evaluation of the primary and secondary hypotheses. Simulations were conducted after completion of the 12 subjects and exclusively based on the residual variance-covariance structure. As the estimated effect size was not taken into account, type 1 error inflation could be neglected. Simulations were performed using SAS 9.4 with a model identical to the analysis model described above. Simulations used the a priori defined minimally relevant effect size of 40% on AUC Pain_3°C_ with variance and correlations estimated from the internal pilot data. Simulations were designed to show the necessary sample size to detect the relevant effect with a power of at least 80%, accepting the 2-sided probability of a type I error of 5%. There was a prespecified decision tree after the internal pilot, approved by the ethics committee. Based on the simulation, 36 volunteers were necessary and provided a power of 84.8% (78.0% for 30 volunteers, Fig. S2c, http://links.lww.com/PAIN/C192). A dropout rate below 6% was assumed, resulting in an approval to include 36 + 3 volunteers.

### 2.11. Exploratory analysis: influence of sex on primary outcome variables

The main effect “sex” and its interaction with injection type were added to the described models in an exploratory fashion.

### 2.12. Exploratory analysis: estimation of a cold pain threshold shift using a nonlinear mixed-effects model

In an exploratory analysis, we employed a nonlinear mixed-effects model to investigate the potential shift in the temperature at which pain is perceived, influenced by various inhibitors. This analysis was conducted using the NLME-package of R, fitting the following specific function to the pain ratings (scaled from 0 to 100) recorded during the cold ramp up to 90 seconds. The model includes a pain response function that remains at zero until reaching a threshold temperature, beyond which the response follows an exponential increase. The strength of the exponential temperature dependence, *b*, and the threshold parameter are estimated from the observed data. The parameter b was introduced as a nuisance parameter to allow a fit to an individually variable suprathreshold stimulus response function.

Data preparation: The temperature data were log_10_-transformed to address potential skewness in the threshold distribution. Consistent with our primary analysis, the insertion pain variable was also log_10_(x + 1)-transformed. A binary dummy variable was created for each type of injection, such as “cold control” and “TRPM8 inhibition.” The model was configured to estimate individual pain response thresholds and the increase in pain beyond the threshold for each subject, treating these 2 parameters as subject-specific random effects. Fixed effects in the model represent the shifts in threshold and change in pain exponent because of each type of injection. The nonlinear mixed-effects model was fitted to capture the individual variability in response, with each subject having their unique threshold and response rate for the control injection, thus taking account of intraindividual correlations. The predicted curves for each injection type were generated and visualized alongside the observed data points to assess model fit visually. It is crucial to interpret these findings as exploratory due to the post hoc nature of this analysis. The shifts in threshold temperature were back-transformed to the original Celsius scale for interpretability and plotted to demonstrate how each inhibitor potentially alters pain perception temperature. The complete R code used for this analysis is provided in the supplementary materials to ensure transparency and reproducibility of the results.

### 2.13. Exploratory analysis: estimation of a cold pain threshold shift using time-to-event analysis

As an alternative threshold value concept, the last 0 rating was defined as the threshold value. Injections whose last score is 0 do not have an event and are therefore censored at the time of the last observation. Using the R package “survival,” a Cox proportional hazards model was used to estimate those time spans by which the inhibitors shift the occurrence of the last 0 ratings within an injection. To account for intraindividual correlations, a frailty term with a gamma distribution was included. The model also included the log_10_(x + 1)-transformed insertion pain. The complete R code used for this analysis is provided in the supplementary materials to ensure transparency and reproducibility of the results.

### 2.14. Exploratory analysis: correlation of cannula insertion pain and AUC Pain_3°C_

To assess correlations between insertion pain and AUC Pain_3°C_ within different injection types, values were transformed as in the primary analysis (AUC Pain_3°C_ square-root, insertion pain log_10_(x + 1)), and Pearson correlation coefficients were calculated separately for each injection type (including familiarization and both study parts).

### 2.15. Randomization

The predefined sequences were randomly drawn from 1 envelope for part 1 and another envelope for part 2 by the person preparing the solutions. There was only 1 copy of each sequence in each envelope. As soon as the envelope was empty, 6 sequences were added again. The randomization was thus carried out in blocks of 6.

## 3. Results

The experimental setup, which is described in detail in the Methods was visualized (Figs. 1A and B).

### 3.1. Participants and recruitment

According to the study protocol, 18 men and 18 women were included. Each individual was randomly assigned to a sequence for part 1, the cold pain model, and to another sequence for part 2, the hypothesis testing (Fig. 1C). All included subjects completed the whole study protocol and were analyzed for the primary and secondary outcomes. Additional analyses were also performed on the full data set (Fig. S2c, http://links.lww.com/PAIN/C192). The first participant was included on July 7, 2023, and the 36th participant on September 1, 2023. There was no follow-up because all outcome variables were obtained within 1 visit. The trial ended when the target sample size of 36 was reached. The age range of participants was 19 to 38 years (female median, 25.0 years, interquartile range, 23.0-27.1 years, male median, 25.0 years, interquartile range, 22.1-28.9 years).

### 3.2. A new human cold pain model

**Figure 2.**
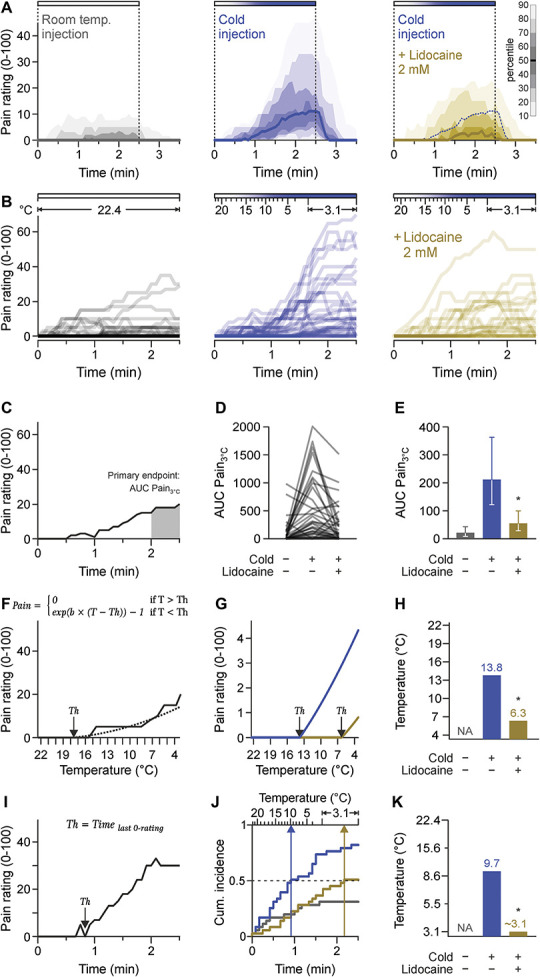
A human cold pain model. (A) Distribution of pain ratings reported every 5 seconds on a 0 to 100 numerical scale upon injection of synthetic interstitial fluid with a constant temperature of 22.4°C, an increasingly cold injection, and an increasingly cold injection containing lidocaine. The horizontal bars at the top represent the periods during which fluid was injected. Data are presented as median (bold line) with shades representing 10% percentiles as indicated by the inset. The median pain ratings of the increasingly cold injection are provided as a visual reference to the panel with the cold injection containing lidocaine. (B) Individual pain rating traces throughout the injection periods in the context of the temperature reached at the cannula tip. (C) Visualization of the primary endpoint (AUC Pain_3°C_) based on a specimen from a cooled injection. (D) Spaghetti plot of the AUC Pain_3°C_ values of all 36 volunteers across the 3 injections. (E) Back-transformed least-squares means with 95% confidence intervals of AUC Pain_3°C_ values. (F) A concept to determine a cold pain threshold, where pain is modeled as a function of temperature (T) with 2 segments. Pain is 0 below the threshold Th; above, it is modeled by a function allowing an individual curvature determined by the parameter b. The presented curve is a representative cold stimulation with the estimated function (dotted line). Pain ratings of all subjects and all injections are provided in Fig. S5 and S6, http://links.lww.com/PAIN/C192. (G) Model-based average temperature-dependent pain ratings for cold injections (blue) and cold injections with lidocaine 2 mM (ocre), and (H) the estimated cold pain thresholds. (I) Cold pain threshold determined by an alternative approach, reporting the time of the last 0 rating. (J) Survival time analysis was applied to these time-to-event data. In the curves showing the cumulative incidence of the threshold, vertical arrows mark the time and the corresponding temperature at which 50% of subjects reached the threshold. (K) Median cold pain thresholds. For room temperature injection, only 11 of 36 (<50%) fulfilled the threshold definition. For the cooled injection, the median cold pain threshold was reached at 9.7°C, the median threshold when lidocaine was coinjected cannot be determined exactly as the time point falls in a time period of a constant temperature of 3.1°C. *P < 0.001.

The first 3 injections were used to confirm that the cold was causing pain. Injection of room temperature interstitial fluid induced marginal levels of pain (Figs. 2A and B), most likely because of local mechanical distension. The pain ratings were higher in cold injections compared with room temperature injections. Adding the positive control lidocaine to increasingly cold fluid reduced the resulting pain ratings (Figs. 2A and B). Cold pain ratings of all subjects are visualized to show variability and to allow for scrutinizing the differences between injections at an individual level (Fig. S5, http://links.lww.com/PAIN/C192). The primary endpoint was AUC Pain_3°C_ and represents an integral measure of pain during the last 30 seconds of the injection protocol, during which the temperature was constant (Fig. 2C). The least squares geometric mean AUC Pain_3°C_ was 212 for cold injections compared with 22 for room temperature injections (*P* < 0.001). This corresponds to an average 9.8-fold increase in pain due to low temperature (95% bootstrap CI 5.4-21.9). Adding lidocaine to cold injections reduced the geometric mean pain to 55.3 (*P* < 0.001 vs cold injections, Figs. 2D and E), which corresponds to a pain reduction by 74% (95% CI 55-86). Because the injections were identical apart from the temperature and lidocaine and were conducted in a blinded manner, it can be inferred that the largest part of pain in this model is indeed caused by cold and that the established model is suitable to detect and quantify pharmacological intervention. For those subjects with a nonzero pain rating at the end of the cooled infusion, pain subsided within 35 seconds (median, range 10-105 seconds). Extending the statistical model by the factor sex did not indicate that AUC Pain_3°C_ differences between the injections were dependent on sex (interaction sex × injection type *P* = 0.73), nor that AUC Pain_3°C_ is generally affected by sex (main effect *P* = 0.28). The predefined secondary endpoint, the area under the curve of pain ratings throughout the whole injection period including the temperature ramp (AUC Pain_Total_), resulted in similar changes. Cold augmented AUC Pain_Total_ values on average by a factor of 8.1 (95% Ci 4.7-15.4, *P* < 0.001), and addition of lidocaine reduced the resulting pain by 75% (95% CI, 57-86, *P* < 0.001). The cold pain model is therefore clearly suitable for the intended purpose on the basis of both the primary and secondary endpoints.

### 3.3. Cold pain thresholds

The temperature ramp has been designed with an emphasis on linearity over a wide temperature range to allow analysis of cold pain thresholds. Two distinct exploratory threshold concepts were applied. First, pain ratings were analyzed using a nonlinear mixed-effects model to directly estimate a threshold. The chosen mathematical function is 0 below the pain threshold and allows an individually bent stimulus response above (Fig. 2F). For the statistical analysis, all pain ratings from all subjects across the 3 injections up to and including 90 seconds, ie, until the target temperature was reached, but excluding the temperature plateau, were used. The resulting estimated thresholds were 13.8°C for the cold injection and 6.3°C for the cold injection with lidocaine (delta 7.5°C, *P* < 0.001, Figs. 2G and H). The second threshold concept is based on the assumption that all positive pain ratings that are followed by a further pain rating of 0 later in the course of the injection, ie, at a lower temperature, are only mechanically induced or represent some noise in the rating. Based on this assumption, the time point at which 0 is rated for the last time was defined as the cold pain threshold for this second threshold concept (Fig. 2I). This procedure produces censored values for those subjects who rated 0 at the end of the injection. These data can therefore be statistically analyzed within the framework of the time-to-event models. From the corresponding cumulative incidence plots, the time points when half of all subjects have reached their threshold can be determined, which is 55 seconds for the cold injection and 130 seconds for the injection with lidocaine (Fig. 2J). The first time point (55 seconds) corresponds to 9.7°C, the second one (130 seconds) falls within a period of constant temperature at 3.1°, which is why it cannot be assigned a specific temperature (Figs. 2J and K). Based on a Cox proportional hazards model, the cold injections with and without lidocaine differ (*P* < 0.001). For the room temperature injection, no comparable pain threshold can be given, as at no time point did 50% of the test subjects reach the defined threshold.

### 3.4. Probing TRPM8 and TRPA1 as cold sensors and Na_V_1.7 and Na_V_1.8 as cold conductors in humans

**Figure 3.**
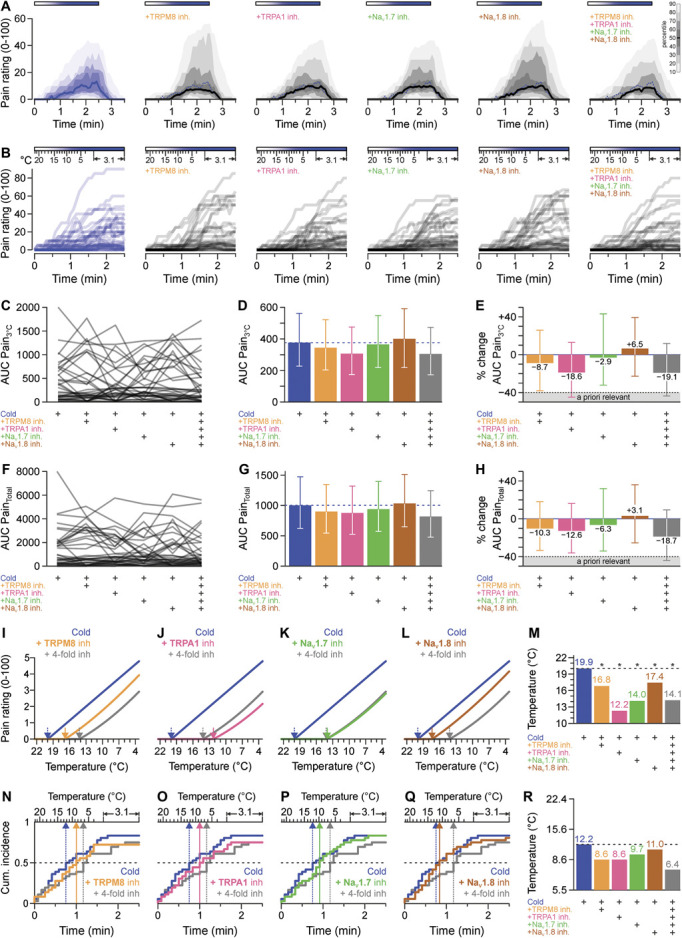
Cold-induced pain ratings in response to pharmacological inhibition of TRPM8, TRPA1, Na_V_1.7, and Na_V_1.8. (A) Each panel shows the distribution of pain ratings at each time point during and after increasingly cold injections indicated by the horizontal bar above the data from 36 subjects. The following inhibitors were added to the injected solutions as indicated: TRPM8 inhibitor PF-05105679 (100 µM), TRPA1 inhibitor A-967079 (10 µM), Na_V_1.7 inhibitor PF-05089771 (1.1 µM), and Na_V_1.8 inhibitor PF-06305591 (1.5 µM). The dotted blue line is the median of the control experiment as visual reference. The distribution of all time points is visualized with the median as a solid line and decreasing blue or gray shades for percentiles more distant from the median in 10% percentile steps indicated by the scale bar (B) Individual pain rating traces throughout the injection periods in the context of coinjected substances and the temperature reached at the cannula tip. (C) Distribution of AUC Pain_3°C_ values, the primary outcome variable. (D) Prespecified primary analysis, reported with back-transformed least-squares means and 95% confidence intervals. (E) Percent changes in AUC Pain_3°C_ values with bootstrapped 95% confidence intervals. The gray area shows the effect size considered a priori as relevant in the sample size calculation. The 95% confidence intervals for TRPA1 inhibition and quadruple inhibition encompass both the null hypothesis and the threshold for a relevant effect (40% inhibition). (F) Distribution of AUC Pain_Total_ values, the secondary outcome variable. (G) Back-transformed least-squares means resulting from the prespecified secondary analysis with 95% confidence intervals. (H) Percent changes in AUC Pain_Total_ values with bootstrapped 95% confidence intervals, which are largely similar to the results of the primary outcome variable. (I-L) Model-based estimated pain ratings over time for each antagonist and their combination. The arrows pointing downward show the temperature of the thresholds estimated from the model. Each panel contains both the control (cold) and the quadruple combination of inhibitors to enable a direct comparison with the respective inhibitors alone. (M) Estimated cold pain thresholds from the model described in panels (I-L). *P < 0.001. (N-Q) Cumulative incidence curves of threshold occurrence (ie, the last 0 rating) for the control (cold) for each inhibitor alone and for their quadruple combination. (R) Summary of median cold pain thresholds derived from cumulative incidence plots. Na_V_1.7, voltage-gated sodium channel, type Na_V_1.7; Na_V_1.8, voltage-gated sodium channel, type Na_V_1.8; TRP, transient receptor potential ion channel, with family and numerator.

Despite the addition of the respective antagonists in the second part, in particular at the end of the cold stimulation, the pain ratings were not substantially different (Figs. 3A and B, Fig. S6, http://links.lww.com/PAIN/C192). There was no evidence that any of the antagonists or the combination of all 4 antagonists reduced cold pain as measured by the primary outcome variable AUC Pain_3°C_ to an extent expected for a principal sensor or a relevant action potential conductor (Figs. 3C and D). Although some AUC Pain_3°C_ values showed a nominal reduction, all 95% confidence intervals for the change in AUC Pain_3°C_ include zero, thus providing no basis to reject the null hypotheses (Fig. 3E). Notably, the 95% confidence interval corresponding to the pain reduction because of TRPA1 inhibition and quadruple inhibition includes also the a priori defined, minimally, clinically relevant, alternative hypothesis of 40% inhibition.

The results for the secondary endpoint were consistent with those of the primary endpoint (Figs. 3F–H). There was a nominal decrease in pain due to the quadruple inhibition by 18.7% and to a lesser extent by the inhibition of channels separately. The 95% confidence interval corresponding to quadruple inhibition also includes both the null hypothesis and 40% reduction. Thus, both the primary and secondary endpoint suggest that a major contribution of the investigated ion channels to cold pain perception is unlikely, although a potential minor role for these channels cannot be ruled out. The statistical test investigating a possible supra- or infra-additivity of the inhibitors resulted in a *P* value of 0.92. Thus, there is no evidence that the quadruple combination results in reductions of pain ratings that are different from the sum of the isolated effects. AUC Pain_3°C_ values were not affected by sex (main effect *P* = 0.54) nor were the effects of inhibitors dependent on sex (interaction sex × injection type *P* = 0.43).

### 3.5. Exploratory analyses of contributions to cold pain threshold

The 2 threshold concepts used to validate the cold pain model were also applied to the hypothesis-testing study part. Direct estimation of the cold pain threshold by the above-described, nonlinear, mixed-effects model resulted in an estimated threshold of 19.9°C for the cold injection. Addition of the TRPM8 inhibitor alone led to an estimated threshold of 8.6°C (Fig. 3I). Addition of TRPA1, Na_V_1.7, or Na_V_1.8 antagonists alone elicited estimated thresholds of 12.2°C (TRPA1 inhibition), 14.0°C (Na_V_1.7 inhibition), and 17.4°C (Na_V_1.8 inhibition, Figs. 3J–L). The combination of the 4 antagonists shifted the threshold to 14.1°C (Fig. 3M). All of the threshold shifts determined in the presence of inhibitors were significant (*P* < 0.001).

In addition, when the cold pain threshold is defined as the last 0 rating given throughout the cold injection, the results are compatible with relevant effects of the inhibitors. Half of the volunteers provided their last 0 rating until 12.2°C. Addition of the TRPM8 inhibitor shifted the corresponding temperature to 8.6°C, respective temperatures for TRPA1, Na_V_1.7, and Na_V_1.8 inhibition were 8.6, 9.7, and 11.0°C. When all 4 inhibitors were combined, half of the volunteers gave their last 0 rating when the temperature of the injected solution had reached 6.4°C (Figs. 3N–R). Statistical comparison of the cumulative incidence curves with the cold control yielded: TRPM8 *P* = 0.16 (Fig. 3N), TRPA1 *P* = 0.07 (Fig. 3O), Na_V_1.7 *P* = 0.36 (Fig. 3P), Na_V_1.8 *P* = 0.44 (Fig. 3Q), quadruple inhibition *P* = 0.058 (not corrected for multiple testing). Despite the fundamental differences in both threshold concepts, both methods indicate a 5.8°C shift in the cold pain threshold with the quadruple inhibition.

### 3.6. Relationship between insertion pain and the outcome variable

In a prior study, insertion pain was associated with the subsequent pain induced by the experimental protocol; this was confirmed in this study (r = 0.13, *P* = 0.019, Fig. S7, http://links.lww.com/PAIN/C192).

### 3.7. Harms

A condition requiring medical attention was not observed in any of the subjects. With abovezero final temperatures for a short period, no negative effects on the tissue were expected and observed.

## 4. Discussion

We established an injection-based human cold pain model that allows testing compounds at the site of cooling. Lidocaine served as positive control and largely inhibited cold pain. In contrast to prior cellular and animal experiments, specific receptor inhibition in humans indicated that neither TRPM8 nor TRPA1 is the principal cold sensor for sustained cold, nor that conduction of cold pain solely relies on Na_V_1.7 or Na_V_1.8. Nevertheless, the data do indicate a role of these channels in cold pain, as the combination of all inhibitors shifted the cold pain threshold by 5.8°C.

To exclude that the substances might not have reached their targets in sufficient concentrations, adsorption to the injection tubing was managed by measuring substances in the cannula outflow and adjusting for their loss. TRPM8 and TRPA1 antagonists completely inhibited the ion channels in vitro using calcium imaging at the concentrations used and at 6-fold lower concentrations, tested due to the previously reported 6-fold higher EC_50_ for intradermal injections compared with cellular experiments.^17^ Cold might spread faster than the injected inhibitors. However, the injected fluid is not yet cold when the injection starts; therefore, the injection site gets presaturated with inhibitors before being cooled. Furthermore, the positive control lidocaine largely inhibited cold pain.

### 4.1. Why the apparent role of the investigated cold sensors might have been limited

In contrast to evidence in favor of the investigated targets, not all studies observed a role for local inhibition of, eg, TRPM8, TRPA1, and Nav1.8 in mouse cold pain models.^37^ In TRPM8-knockout mice, cooling perception around 25°C is absent. However, at temperatures approaching 5°C, after ablating the TRPM8-positive neuron population, behavioral responses to cold were further reduced compared with TRPM8-knockout mice, but definitely not abolished, suggesting a neuronal population responsible for cold perception only partly overlapping with TRPM8-positive neurons.^23^ A meta-analysis confirmed that TRPM8 agonist menthol increased the cold pain threshold.^45^ However, the largest study had censored data in 50 of 125 subjects, almost entirely from cold pain detection, excluding these from analysis.^14^ The cold pressor test assesses cold pain tolerance and produces much higher pain intensities than the model proposed here, which may limit comparability. Nevertheless, systemic administration of the TRPM8 antagonist PF-05105679 reduced pain in the cold pressor test in healthy subjects.^48^ However, as only the difference to placebo but not the group means were reported, the effect size cannot be assessed, rendering a comparison with our results impossible. The corresponding author did not reply upon request. TRPM8 antagonist RQ-00434739^1^ is currently tested in a phase I trial (ACTRN12624000245594).

TRPA1's controversial role in cold pain has been extensively reviewed.^24,41,43,50^ TRPA1 agonist applications do not allow easy conclusions, as stimulation can desensitize,^2^ but also sensitize TRPA1,^11,31^ which would lead to opposing effects. Most TRPA1 agonists have low potency, which do not favor specificity. Topical cinnamaldehyde application reduced cold pain thresholds slightly more than vehicle,^35^ but not in 2 other studies.^36,46^ Intradermal injection of a specific TRPA1 agonist did not induce cold sensation.^17^ TRPA1 antagonist LY3526318 applied orally reduced cinnamaldehyde-induced blood flow increase in humans, with no cold-related outcomes being reported.^4^ The effects of TRPA1 antagonist ISC 17536 (GRC 17536) were evaluated weekly by quantitative sensory testing as secondary endpoint. The lack of reporting changes of cold pain thresholds in this trial might be seen as indirect negative evidence.^21^

For Na_V_1.7, with a limited choice of inhibitors,^12^ PF-05089771 was considered the best option regarding potency and selectivity. Despite positive studies, there are also studies reporting limited efficacy. In 24 subjects tested in several human pain models, including a cold pressor test,^40^ there was no evidence for an analgesic effect of 300 mg PF-05089771 orally. Furthermore, a study with 1600-mg oral dose in 5 erythromelalgia patients with a gain-of-function mutation in Na_V_1.7 was not decisive.^9^ Their reported median plasma concentration 4 and 6 hours after ingestion were above 100 µM, which is about 90-fold of our injected concentration. Na_V_1.7 pharmacology is known to be challenging, as a large number of Na_V_1.7 inhibitors with promising in vitro and in animal model results failed in humans.^12^

The outlined pivotal role of Na_V_1.8 for cold sensing in preclinical data^52^ was challenged by similar cold responsiveness in Na_V_1.8-positive and Na_V_1.8-negative sensory neurons.^26^ A role only below 0°C has been suggested.^37^ The potent Na_V_1.8 inhibitor VX-150 did not influence heat-induced, pressure-induced or electrically induced pain in healthy subjects but substantially prolonged cold pain tolerance in the cold pressor test for several hours after oral dosing.^19^ However, the cold pressor test retraction times indicate that other mechanisms still cause pain beyond tolerance. VX-150 is a prodrug, and conversion in the skin to the active form would need be clarified before testing it in our cold pain model. The Na_V_1.8 inhibitor suzetrigine (VX-548), which has completed 3 clinical phase III trials, has become commercially available after this study's approval. Whether the data from our study are sufficient to exclude Na_V_1.8 as a major conductor of cold pain is questionable, but in synopsis with published studies, Na_V_1.8 is unlikely to be the sole conductor.

### 4.2. Exploratory results

The exploratory analyses focused on cold pain thresholds with 2 distinct threshold concepts; both have advantages and disadvantages. The first threshold concept fitted a predefined mathematical function to all raw pain scores and has the statistical advantage of using all ratings without prior aggregation and modelling suprathreshold pain time courses. This type of modeling requires several assumptions, including the selection of the function, the selection of random effects, or the necessity of data transformations. This model showed a shift in the cold pain threshold towards lower temperatures for all 4 targets. The largest single threshold shift was observed for inhibition of TRPA1. This is remarkable because there was no prior human data in favor of a TRPA1 contribution to cold pain. Above the individual thresholds, the shift of the suprathreshold stimulus–response curve by all antagonists appeared largely parallel to the native sensitivity. This could be interpreted as multiple sensors detecting cold, and their suprathreshold contribution being largely constant. Below the temperature threshold, a receptor is responsible for a constant shift towards lower temperature. From such a model, one should expect that the inhibition of independent sensors has an at least to some degree additive effect, and it is puzzling that this was not observed in the quadruple combination of antagonists. At increasingly lower temperatures, the fraction of cold pain not explained by the 4 receptors is growing. This culminates at the end of the prolonged cold stimulus, where pain was altered by none of the antagonists or their quadruple combination to an extent that would justify the conclusion that a major cold sensor or conductor had been blocked. The second threshold concept reduces each individual pain rating trace to a summary measure, the time point of the last 0 rating. This requires fewer statistical assumptions, rendering this threshold concept more generalizable, but sacrifices all available suprathreshold information. It should be emphasized that despite these differences in approach, both analyses resulted in a threshold shift of 5.8°C because of quadruple inhibition.

Further candidate molecules for cold pain have been the topic of several topical reviews.^8,44,45^ Briefly, besides a direct activation of cold-gated ion channels, including voltage-gated sodium and calcium channels, activation of the glutamate receptor GluK2 or STIM/Orai and also inhibition of voltage-gated potassium channels and 2-pore-domain potassium channels, or the Na^+^/K^+^-ATPase was suggested.

### 4.3. Limitations

Our study was conducted on skin; no other sites were tested. The study addressed physiological mechanisms in healthy subjects, which may differ in patients with cold allodynia and cold hyperalgesia, eg, after oxaliplatin treatment. This involves structural damage to nerve fibers, which causes substantial alterations to nerve excitability.^10^ The alterations are at least partially selective regarding fiber type,^5^ which could shift the balance in sensory inputs.

Cold temperatures evoke distinct sensations, ranging from mild unpleasantness to intense pain. Considering unpleasantness as criterion has led to reports in which the cold pain threshold is above 25°C in 50% of subjects.^47^

The tested antagonists are lipophilic, affecting their rate constants to and from lipophilic compartments, which could hinder convection and diffusion. There is indirect evidence from redistribution of hydrophilic and lipophilic dyes in transdermal assays, although the results from the epidermis might not translate to intradermal tissue.^20^ The xLogP3 values of the applied inhibitors for TRPA1, TRPM8, Na_V_1.7, and Na_v_1.8 are 3.2, 4.7, 4.3, and 1.5, respectively. Lidocaine, with an xLogP3-value of 2.3, also reduced pain. Also, the effectiveness of TRPA1 inhibitor A-967079 against pain in humans argues against false-negative results because of lipophilicity.^17^

## 5. Conclusion

TRPA1 and TRPM8 most likely contribute to cold pain, whereas Na_V_1.7 and Na_v_1.8 probably do not. Yet, they certainly do not fully explain cold pain. Thus, at least 1 important additional sensor for cold exists in humans.

## Conflict of interest statement

The authors have no conflicts of interest to declare.

## Appendix A. Supplemental digital content

Supplemental digital content associated with this article can be found online at http://links.lww.com/PAIN/C192.

## Supplementary Material

SUPPLEMENTARY MATERIAL

## References

[1] Aizawa N Ohshiro H Watanabe S Kume H Homma Y Igawa Y. RQ-00434739, a novel TRPM8 antagonist, inhibits prostaglandin E2-induced hyperactivity of the primary bladder afferent nerves in rats. Life Sci 2019;218:89–95. 30580018 10.1016/j.lfs.2018.12.031

[2] Akopian AN Ruparel NB Jeske NA Hargreaves KM. Transient receptor potential TRPA1 channel desensitization in sensory neurons is agonist dependent and regulated by TRPV1-directed internalization. J Physiol 2007;583:175–93. 17584831 10.1113/jphysiol.2007.133231 PMC2277224

[3] Alexandrou AJ Brown AR Chapman ML Estacion M Turner J Mis MA Wilbrey A Payne EC Gutteridge A Cox PJ Doyle R Printzenhoff D Lin Z Marron BE West C Swain NA Storer RI Stupple PA Castle NA Hounshell JA Rivara M Randall A Dib-Hajj SD Krafte D Waxman SG Patel MK Butt RP Stevens EB. Subtype-selective small molecule inhibitors reveal a fundamental role for Nav1.7 in nociceptor electrogenesis, axonal conduction and presynaptic release. PLoS One 2016;11:e0152405. 27050761 10.1371/journal.pone.0152405 PMC4822888

[4] Bamps D Blockeel AJ Dreesen E Marynissen H Laenen J Van Hecken A Wilke A Shahabi S Johnson KW Collins EC Broad LM Phillips KG de Hoon J. TRPA1 antagonist LY3526318 inhibits the cinnamaldehyde-evoked dermal blood flow increase: translational proof of pharmacology. Clin Pharmacol Ther 2023;114:1093–103. 37562824 10.1002/cpt.3024

[5] Bennedsgaard K Ventzel L Andersen NT Themistocleous AC Bennett DL Jensen TS Tankisi H Finnerup NB. Oxaliplatin- and docetaxel-induced polyneuropathy: clinical and neurophysiological characteristics. J Peripher Nerv Syst 2020;25:377–87. 32902058 10.1111/jns.12413 PMC7756561

[6] Bretag AH. Synthetic interstitial fluid for isolated mammalian tissue. Life Sci 1969;8:319–29. 10.1016/0024-3205(69)90283-5 5781321

[7] Brown AD Bagal SK Blackwell P Blakemore DC Brown B Bungay PJ Corless M Crawforth J Fengas D Fenwick DR Gray V Kemp M Klute W Malet Sanz L Miller D Murata Y Payne CE Skerratt S Stevens EB Warmus JS. The discovery and optimization of benzimidazoles as selective Na(V)1.8 blockers for the treatment of pain. Bioorg Med Chem 2019;27:230–9. 30538065 10.1016/j.bmc.2018.12.002

[8] Buijs TJ McNaughton PA. The role of cold-sensitive ion channels in peripheral thermosensation. Front Cell Neurosci 2020;14:262. 32973456 10.3389/fncel.2020.00262 PMC7468449

[9] Cao L McDonnell A Nitzsche A Alexandrou A Saintot PP Loucif AJ Brown AR Young G Mis M Randall A Waxman SG Stanley P Kirby S Tarabar S Gutteridge A Butt R McKernan RM Whiting P Ali Z Bilsland J Stevens EB. Pharmacological reversal of a pain phenotype in iPSC-derived sensory neurons and patients with inherited erythromelalgia. Sci Transl Med 2016;8:335ra56. 10.1126/scitranslmed.aad7653 27099175

[10] Chiorazzi A Canta A Carozzi VA Meregalli C Pozzi E Ballarini E Rodriguez-Menendez V Marmiroli P Cavaletti G Alberti P. Morphofunctional characterisation of axonal damage in different rat models of chemotherapy-induced peripheral neurotoxicity: the role of nerve excitability testing. J Peripher Nerv Syst 2024;29:47–57. 38009865 10.1111/jns.12607

[11] del Camino D Murphy S Heiry M Barrett LB Earley TJ Cook CA Petrus MJ Zhao M D'Amours M Deering N Brenner GJ Costigan M Hayward NJ Chong JA Fanger CM Woolf CJ Patapoutian A Moran MM. TRPA1 contributes to cold hypersensitivity. J Neurosci 2010;30:15165–74. 21068322 10.1523/JNEUROSCI.2580-10.2010 PMC3021322

[12] Eagles DA Chow CY King GF. Fifteen years of Na(V) 1.7 channels as an analgesic target: why has excellent in vitro pharmacology not translated into in vivo analgesic efficacy? Br J Pharmacol 2022;179:3592–611. 33206998 10.1111/bph.15327

[13] Emery EC Luiz AP Wood JN. Nav1.7 and other voltage-gated sodium channels as drug targets for pain relief. Expert Opin Ther Targets 2016;20:975–83. 26941184 10.1517/14728222.2016.1162295 PMC4950419

[14] Fluhr K Neddermeyer TJ Lotsch J. Capsaicin or menthol sensitization induces quantitative but no qualitative changes to thermal and mechanical pain thresholds. Clin J Pain 2009;25:128–31. 19333158 10.1097/AJP.0b013e3181817aa2

[15] Goodwin G McMahon SB. The physiological function of different voltage-gated sodium channels in pain. Nat Rev Neurosci 2021;22:263–74. 33782571 10.1038/s41583-021-00444-w

[16] Heber S Ciotu CI Hartner G Gold-Binder M Ninidze N Gleiss A Kress HG Fischer MJM. TRPV1 antagonist BCTC inhibits pH 6.0-induced pain in human skin. PAIN 2020;161:1532–41. 32107360 10.1097/j.pain.0000000000001848

[17] Heber S Gold-Binder M Ciotu CI Witek M Ninidze N Kress HG Fischer MJM. A human TRPA1-specific pain model. J Neurosci 2019;39:3845–55. 30862667 10.1523/JNEUROSCI.3048-18.2019 PMC6520506

[18] Heber S Resch F Ciotu CI Gleiss A Heber UM Macher-Beer A Bhuiyan S Gold-Binder M Kain R Sator S Fischer MJM. Human heat sensation: a randomized crossover trial. Sci Adv 2024;10:eado3498. 39231217 10.1126/sciadv.ado3498 PMC11373589

[19] Hijma HJ Siebenga PS de Kam ML Groeneveld GJ. A phase 1, randomized, double-blind, placebo-controlled, crossover study to evaluate the pharmacodynamic effects of VX-150, a highly selective NaV1.8 inhibitor, in healthy male adults. Pain Med 2021;22:1814–26. 33543763 10.1093/pm/pnab032 PMC8346919

[20] Jacobi U Tassopoulos T Surber C Lademann J. Cutaneous distribution and localization of dyes affected by vehicles all with different lipophilicity. Arch Dermatol Res 2006;297:303–10. 16292655 10.1007/s00403-005-0621-5

[21] Jain SM Balamurugan R Tandon M Mozaffarian N Gudi G Salhi Y Holland R Freeman R Baron R. Randomized, double-blind, placebo-controlled trial of ISC 17536, an oral inhibitor of transient receptor potential ankyrin 1, in patients with painful diabetic peripheral neuropathy: impact of preserved small nerve fiber function. PAIN 2022;163:e738–47. 34490850 10.1097/j.pain.0000000000002470 PMC9100440

[22] Kim H Mittal DP Iadarola MJ Dionne RA. Genetic predictors for acute experimental cold and heat pain sensitivity in humans. J Med Genet 2006;43:e40. 16882734 10.1136/jmg.2005.036079 PMC2564596

[23] Knowlton WM Palkar R Lippoldt EK McCoy DD Baluch F Chen J McKemy DD. A sensory-labeled line for cold: TRPM8-expressing sensory neurons define the cellular basis for cold, cold pain, and cooling-mediated analgesia. J Neurosci 2013;33:2837–48. 23407943 10.1523/JNEUROSCI.1943-12.2013 PMC3711390

[24] Laursen WJ Anderson EO Hoffstaetter LJ Bagriantsev SN Gracheva EO. Species-specific temperature sensitivity of TRPA1. Temperature (Austin) 2015;2:214–26. 27227025 10.1080/23328940.2014.1000702 PMC4843866

[25] Lotsch J Dimova V Lieb I Zimmermann M Oertel BG Ultsch A. Multimodal distribution of human cold pain thresholds. PLoS One 2015;10:e0125822. 25992576 10.1371/journal.pone.0125822 PMC4439151

[26] Luiz AP MacDonald DI Santana-Varela S Millet Q Sikandar S Wood JN Emery EC. Cold sensing by Na(V)1.8-positive and Na(V)1.8-negative sensory neurons. Proc Natl Acad Sci U S A 2019;116:3811–6. 30755524 10.1073/pnas.1814545116 PMC6397562

[27] Luo MH Wang Z Zhang H Arens E Filingeri D Jin L Ghahramani A Chen WH He YD Si BH. High-density thermal sensitivity maps of the human body. Building Environ 2020;167:106435.

[28] Magerl W Krumova EK Baron R Tolle T Treede RD Maier C. Reference data for quantitative sensory testing (QST): refined stratification for age and a novel method for statistical comparison of group data. PAIN 2010;151:598–605. 20965658 10.1016/j.pain.2010.07.026

[29] Maier C Baron R Tolle TR Binder A Birbaumer N Birklein F Gierthmuhlen J Flor H Geber C Huge V Krumova EK Landwehrmeyer GB Magerl W Maihofner C Richter H Rolke R Scherens A Schwarz A Sommer C Tronnier V Uceyler N Valet M Wasner G Treede DR. Quantitative sensory testing in the German Research Network on Neuropathic Pain (DFNS): somatosensory abnormalities in 1236 patients with different neuropathic pain syndromes. PAIN 2010;150:439–50. 20627413 10.1016/j.pain.2010.05.002

[30] McDermott LA Weir GA Themistocleous AC Segerdahl AR Blesneac I Baskozos G Clark AJ Millar V Peck LJ Ebner D Tracey I Serra J Bennett DL. Defining the functional role of Na(V)1.7 in human nociception. Neuron 2019;101:905–19.e8. 30795902 10.1016/j.neuron.2019.01.047 PMC6424805

[31] Meents JE Fischer MJ McNaughton PA. Agonist-induced sensitisation of the irritant receptor ion channel TRPA1. J Physiol 2016;594:6643–60. 27307078 10.1113/JP272237 PMC5108891

[32] Middleton SJ Perini I Themistocleous AC Weir GA McCann K Barry AM Marshall A Lee M Mayo LM Bohic M Baskozos G Morrison I Loken LS McIntyre S Nagi SS Staud R Sehlstedt I Johnson RD Wessberg J Wood JN Woods CG Moqrich A Olausson H Bennett DL. Nav1.7 is required for normal C-low threshold mechanoreceptor function in humans and mice. Brain 2022;145:3637–53. 34957475 10.1093/brain/awab482 PMC9586547

[33] Minett MS Nassar MA Clark AK Passmore G Dickenson AH Wang F Malcangio M Wood JN. Distinct Nav1.7-dependent pain sensations require different sets of sensory and sympathetic neurons. Nat Commun 2012;3:791. 22531176 10.1038/ncomms1795 PMC3337979

[34] Moparthi L Kichko TI Eberhardt M Hogestatt ED Kjellbom P Johanson U Reeh PW Leffler A Filipovic MR Zygmunt PM. Human TRPA1 is a heat sensor displaying intrinsic U-shaped thermosensitivity. Sci Rep 2016;6:28763. 27349477 10.1038/srep28763 PMC4923899

[35] Namer B Seifert F Handwerker HO Maihofner C. TRPA1 and TRPM8 activation in humans: effects of cinnamaldehyde and menthol. Neuroreport 2005;16:955–9. 15931068 10.1097/00001756-200506210-00015

[36] Olsen RV Andersen HH Moller HG Eskelund PW Arendt-Nielsen L. Somatosensory and vasomotor manifestations of individual and combined stimulation of TRPM8 and TRPA1 using topical L-menthol and trans-cinnamaldehyde in healthy volunteers. Eur J Pain 2014;18:1333–42. 24664788 10.1002/j.1532-2149.2014.494.x

[37] Patel R Brice NL Lewis RJ Dickenson AH. Ionic mechanisms of spinal neuronal cold hypersensitivity in ciguatera. Eur J Neurosci 2015;42:3004–11. 26454262 10.1111/ejn.13098 PMC4744673

[38] Raja SN Carr DB Cohen M Finnerup NB Flor H Gibson S Keefe FJ Mogil JS Ringkamp M Sluka KA Song XJ Stevens B Sullivan MD Tutelman PR Ushida T Vader K. The revised International Association for the Study of Pain definition of pain: concepts, challenges, and compromises. PAIN 2020;161:1976–82. 32694387 10.1097/j.pain.0000000000001939 PMC7680716

[39] Ruff RL. Effects of temperature on slow and fast inactivation of rat skeletal muscle Na(+) channels. Am J Physiol 1999;277:C937–47. 10564086 10.1152/ajpcell.1999.277.5.C937

[40] Siebenga P van Amerongen G Hay JL McDonnell A Gorman D Butt R Groeneveld GJ. Lack of detection of the analgesic properties of PF-05089771, a selective Na(v) 1.7 inhibitor, using a battery of pain models in healthy subjects. Clin Transl Sci 2020;13:318–24. 31642607 10.1111/cts.12712 PMC7070789

[41] Sinica V Vlachova V. Transient receptor potential ankyrin 1 channel: an evolutionarily tuned thermosensor. Physiol Res 2021;70:363–81. 33982589 10.33549/physiolres.934697 PMC8820563

[42] Stevens JC Choo KK. Temperature sensitivity of the body surface over the life span. Somatosens Mot Res 1998;15:13–28. 9583574 10.1080/08990229870925

[43] Viana F. TRPA1 channels: molecular sentinels of cellular stress and tissue damage. J Physiol 2016;594:4151–69. 27079970 10.1113/JP270935 PMC4967735

[44] Wang H Siemens J. TRP ion channels in thermosensation, thermoregulation and metabolism. Temperature (Austin) 2015;2:178–87. 27227022 10.1080/23328940.2015.1040604 PMC4843888

[45] Weyer-Menkhoff I Lotsch J. Human pharmacological approaches to TRP-ion-channel-based analgesic drug development. Drug Discov Today 2018;23:2003–12. 29969684 10.1016/j.drudis.2018.06.020

[46] Weyer-Menkhoff I Lotsch J. TRPA1 sensitization produces hyperalgesia to heat but not to cold stimuli in human volunteers. Clin J Pain 2019;35:321–7. 30664549 10.1097/AJP.0000000000000677

[47] Weyer-Menkhoff I Pinter A Schlierbach H Schanzer A Lotsch J. Epidermal expression of human TRPM8, but not of TRPA1 ion channels, is associated with sensory responses to local skin cooling. PAIN 2019;160:2699–709. 31343541 10.1097/j.pain.0000000000001660

[48] Winchester WJ Gore K Glatt S Petit W Gardiner JC Conlon K Postlethwaite M Saintot PP Roberts S Gosset JR Matsuura T Andrews MD Glossop PA Palmer MJ Clear N Collins S Beaumont K Reynolds DS. Inhibition of TRPM8 channels reduces pain in the cold pressor test in humans. J Pharmacol Exp Ther 2014;351:259–69. 25125580 10.1124/jpet.114.216010

[49] Winter Z Gruschwitz P Eger S Touska F Zimmermann K. Cold temperature encoding by cutaneous TRPA1 and TRPM8-carrying fibers in the mouse. Front Mol Neurosci 2017;10:209. 28713241 10.3389/fnmol.2017.00209 PMC5492152

[50] Zhang H Wang C Zhang K Kamau PM Luo A Tian L Lai R. The role of TRPA1 channels in thermosensation. Cell Insight 2022;1:100059. 37193355 10.1016/j.cellin.2022.100059 PMC10120293

[51] Zhao M Isami K Nakamura S Shirakawa H Nakagawa T Kaneko S. Acute cold hypersensitivity characteristically induced by oxaliplatin is caused by the enhanced responsiveness of TRPA1 in mice. Mol Pain 2012;8:55. 22839205 10.1186/1744-8069-8-55 PMC3495669

[52] Zimmermann K Leffler A Babes A Cendan CM Carr RW Kobayashi J Nau C Wood JN Reeh PW. Sensory neuron sodium channel Nav1.8 is essential for pain at low temperatures. Nature 2007;447:855–8. 17568746 10.1038/nature05880

